# UK consensus statement on the diagnosis of inducible laryngeal obstruction in light of the COVID‐19 pandemic

**DOI:** 10.1111/cea.13745

**Published:** 2020-10-08

**Authors:** Jemma Haines, Karen Esposito, Claire Slinger, Nicola Pargeter, Jennifer Murphy, Julia Selby, Kathryn Prior, Adel Mansur, Aashish Vyas, Andrew E. Stanton, Ian Sabroe, James H. Hull, Stephen J. Fowler

**Affiliations:** ^1^ Division of Infection Immunity & Respiratory Medicine School of Biological Sciences Faculty of Biology, Medicine and Health The University of Manchester Manchester UK; ^2^ Manchester Academic Health Science Centre and NIHR Manchester Biomedical Research Centre Manchester University Hospitals NHS Foundation Trust Manchester UK; ^3^ Sheffield Teaching Hospitals NHS Foundation Trust Sheffield UK; ^4^ Lancashire Chest Centre Royal Preston Hospital Lancashire Teaching Hospitals NHS Foundation Trust Preston UK; ^5^ Heartlands Hospital University Hospitals Birmingham Birmingham UK; ^6^ Newcastle Upon Tyne Hospitals Newcastle Upon Tyne UK; ^7^ Royal Brompton Hospital London UK; ^8^ Institute of Sport, Exercise and Health University College London London UK; ^9^ English Institute of Sport London UK

**Keywords:** COVID‐19, inducible laryngeal obstruction, laryngoscopy

## Abstract

Prior to the COVID‐19 pandemic, laryngoscopy was the mandatory gold standard for the accurate assessment and diagnosis of inducible laryngeal obstruction. However, upper airway endoscopy is considered an aerosol‐generating procedure in professional guidelines, meaning routine procedures are highly challenging and the availability of laryngoscopy is reduced. In response, we have convened a multidisciplinary panel with broad experience in managing this disease and agreed a recommended strategy for presumptive diagnosis in patients who cannot have laryngoscopy performed due to pandemic restrictions. To maintain clinical standards whilst ensuring patient safety, we discuss the importance of triage, information gathering, symptom assessment and early review of response to treatment. The consensus recommendations will also be potentially relevant to other future situations where access to laryngoscopy is restricted, although we emphasize that this investigation remains the gold standard.

## 1 BACKGROUND

### 1.1 Inducible laryngeal obstruction

Inducible laryngeal obstruction (ILO) is defined as, “inappropriate laryngeal closure at the glottic and/or supraglottic level, which leads to dynamic airflow obstruction and causes breathing difficulties”.^1^, ^2^ Individuals with ILO present across varied healthcare settings with differing levels of morbidity.^3^ Presentation ranges from mild dyspnoea to acute respiratory distress which in severe cases may lead to intubation and mechanical ventilation; symptoms are typically episodic, during inspiration and sudden in onset following exposure to certain triggers.^3^, ^4^, ^5^

These symptoms lead to many individuals being misdiagnosed with asthma, both acutely and in the community.^6^ Healthcare utilization and escalating pharmacological burden, when symptoms do not respond to standard therapies, are common. Further, ILO and asthma can co‐exist, with up to 50% of people with asthma demonstrating abnormal laryngeal movements during respiration.^7^, ^8^

### 1.2 Diagnostics pre‐COVID‐19

The diagnosis of ILO begins with a thorough evaluation of the clinical history. Typical clinical symptoms [eg difficulty breathing in, throat tightness/discomfort, voice disturbance ^1^, ^3^] may raise suspicion of ILO. Other clinical features suggestive of exercise‐ILO (EILO), include symptom onset at high‐intensity exercise, accompanied by stridor and rapid resolution on exercise cessation.^9^, ^10^ However, these features when obtained from clinical history alone have poor diagnostic precision, and as such should be interpreted with caution. Moreover, many symptoms (eg coughing and noisy breathing) are also recognized in other disease states, especially asthma. Therefore, symptoms must be assessed in conjunction with objective test results.

Flexible fibreoptic laryngoscopy during a symptomatic episode is the gold standard for ILO diagnosis.^4^, ^11^ As the upper airway will often appear functionally normal outside of an ILO episode, continuous laryngoscopy during provocation (ie a trigger known to induce ILO symptoms such as inhaled irritants) is often performed.^12^

### 1.3 Changes to practice in the COVID‐19 pandemic

The impact of the COVID‐19 pandemic upon healthcare provision has been profound globally. In the UK, most respiratory elective activity was suspended in March 2020. Hospital‐based respiratory clinicians continue to manage acute COVID‐19 patients whilst, at the same time, attempting to coordinate a safe return to elective services.^13^ To reduce the risk of COVID‐19 infection spreading, services across the UK are urgently reconfiguring to minimize face‐to‐face consultations; many are utilizing telehealth where appropriate.

Upper airway endoscopy has the potential to generate aerosols through sneezing and coughing;^14^, ^15^ in national professional guidelines,^15^, ^16^, ^17^ it is considered a potential aerosol‐generating procedure. Further, the nose and nasopharynx are reservoirs for high concentrations of COVID‐19 virus ^18^ meaning clinicians performing laryngoscopy are at high risk of COVID‐19 exposure. In response, laryngoscopy in the UK from March 2020 was restricted to emergency ENT and cancer cases only, and all speech and language therapy (SLT) led endoscopy ceased.^15^, ^17^ More recently, collaborative professional body guidelines ^19^ outlined a graded return to laryngoscopy and therefore it is anticipated routine work will begin to resume. However, the availability of laryngoscopy will likely remain restricted due to COVID‐19 infection prevention and control guidelines and staff/ patient availability.

Internationally, the impact of COVID‐19 pandemic remains a highly variable situation. As the pandemic evolves, the accessibility to endoscopic evaluation of the larynx is likely to be scaled according to pandemic severity and the rate of community transmissions within geographical locations. Further, the resources available (eg personal protective equipment, COVID‐19 screening) and workforce logistics (eg suitable rooms for procedures, redeployment of staff) will have significant impact on the delivery of care.

### 1.4 Clinical approach to ILO in context

A multidisciplinary team (MDT) approach improves diagnostic precision and facilitates a meaningful clinical impact in ILO.^20^ However, in the absence of routine access to laryngoscopy, a critical component for MDT management of suspected ILO patients is missing, that is the gold‐standard diagnostic tool.

The inability to perform an endoscopic evaluation of the larynx and/or continuous laryngoscopy during provocation limits the MDT’s ability to:

- accurately confirm or refute ILO diagnosis
- directly assess structure and function in the upper airway and rule out mimics that include serious structural issues requiring immediate surgical intervention (eg subglottic stenosis).
- assess relevant differential diagnoses (especially nasal and reflux disease)
- inform treatment regimens specific to the individual patient
- provide biofeedback (to the patient) and teaching
- monitor therapeutic response

Clinical symptoms can be used only as a guide, and without usual access to objective testing, it is not possible to confirm an accurate diagnosis. Over‐diagnosis without laryngoscopy is a particular concern in this context. In 303 patients with a high clinical suspicion of ILO (after specialist MDT evaluation), we confirmed laryngoscopic appearances in only 74% (*data on file*). It may be that symptom‐based questionnaires such as the Pittsburgh Vocal Cord Dysfunction Index^21^ can be used to help select patients, although further validation is needed.^22^ Such an approach has significant implications for ILO management, which should be considered carefully to minimize risk.

As elective activity and procedures are progressively reintroduced, professional guidelines^19^ support service recovery plans. However, specific guidance on how to diagnose ILO in the context of COVID‐19 currently does not exist. In response, This consensus statement aims to guide clinicians working with ILO patient populations.

### 1.5 Methodology

The MDT consensus group (n = 13), represents six UK specialist centres, all of which have established MDT provision for patients with ILO and airways disease. An adaptation of the nominal group technique^23^ was applied for the consensus method: i) lead author sent key questions for consideration in advance of initial virtual meeting; ii) round‐Robin of individual's reflections (virtual meeting); iii) group themes generated for inclusion (virtual meeting); and iv) clarification of content and structure (virtual meeting).

To manage any potential dissention during the statement production, a pre‐determined level of what would constitute consensus agreement was discussed at the outset. However, no such dissention occurred. This statement constitutes the expression of general opinion of all the group experts.

## 2 RECOMMENDATIONS FOR CLINICAL DECISION‐MAKING

### 2.1 Overview

The profound reduction of access to laryngoscopy has necessitated the consideration of alternative approaches to ILO diagnostic and treatment pathways. Other instrumental assessments may be beneficial to detect ILO, including spirometry and dynamic computerized tomography (CT) scanning.^8^ However, in the absence of robust validation of such techniques, they are not recommended for use in routine clinical practice. Further, accessibility to such assessments in the COVID‐19 era is restricted.^24^

**Figure 1:**
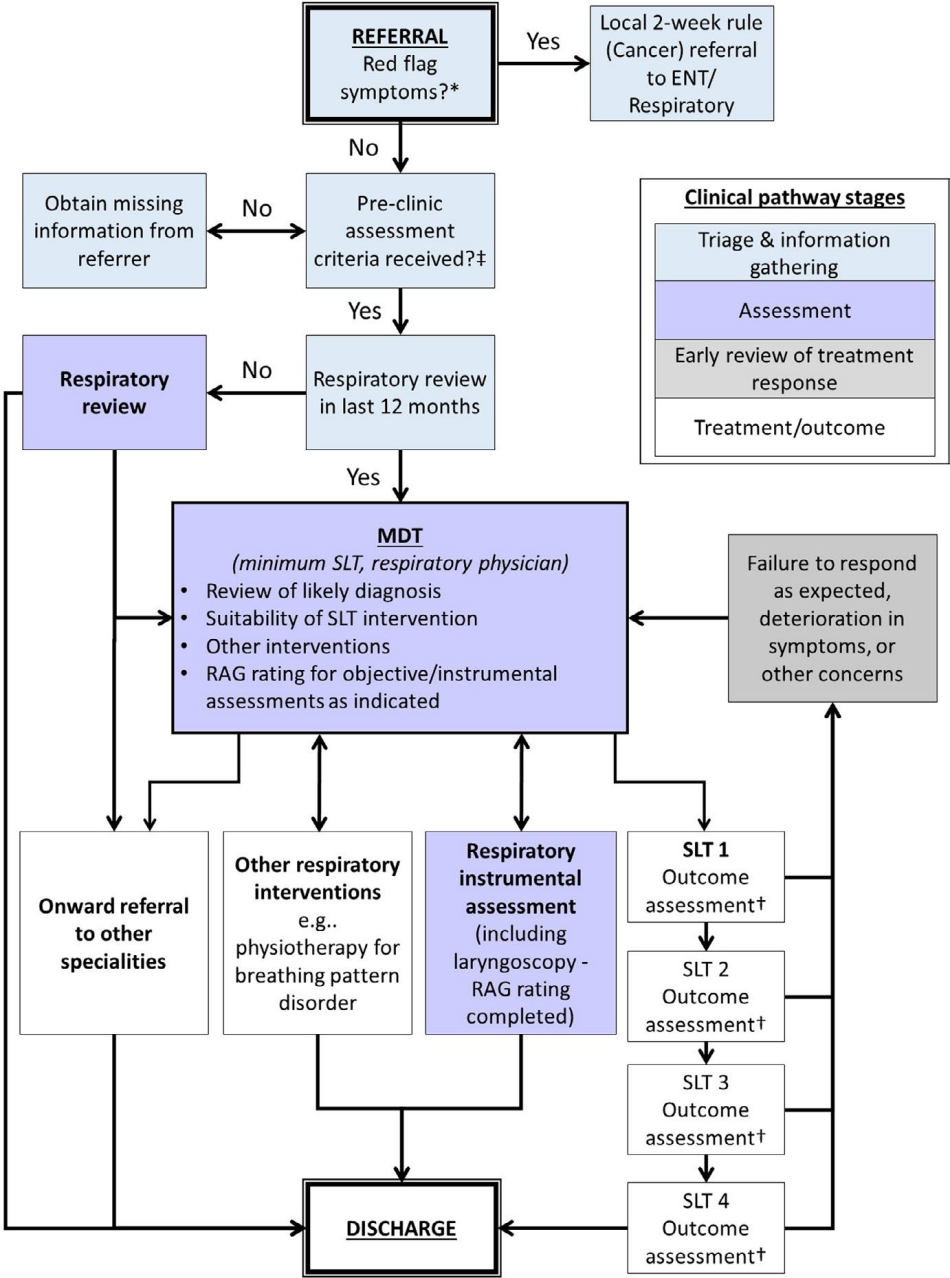
Clinical pathway for suspected inducible laryngeal obstruction in light of COVID‐19. *For “red flag” symptoms see text; †for example clinical symptoms/Vocal Cord Dysfunction Questionnaire/visual analogue scale; ‡ see Table 1. Abbreviations: ENT—ear nose and throat; MDT—multidisciplinary team; SLT—speech and language therapy; RAG—red/amber/green risk assessment, see Table 2

A pragmatic approach to the assessment of ILO is therefore important to minimize ILO morbidity where possible. Diagnosis must rely heavily on clinical clues and be guided by results of previous investigations in order to exclude other causes of symptoms, primarily breathlessness. To minimize risk of potential misdiagnosis (both under‐ and over‐diagnosis), we recommend the following:

- All patients should be managed through a dedicated MDT (minimum requirement SLT and respiratory physician, both with experience managing ILO and related conditions; it is highly recommended to also include a specialist respiratory physiotherapist with experience in managing breathing pattern disorders);
- Management decisions should be guided by the consensus recommended clinical pathway (Figure 1);
- “Suspected ILO” is the term given until an endoscopic evaluation is available to give a confirmatory ILO diagnostic label.

### 2.2 Clinical pathway

The overall aim of the clinical pathway (Figure 1) is to support patient safety whilst enabling identification or exclusion of ILO as appropriate. It is essential that robust local data‐collection procedures are in place to ensure effective monitoring of patients through the pathway. It is expected that in some cases a suspected ILO diagnosis will be incorrect, and so we suggest mitigating this by careful exclusion and/or treatment of co‐morbidities during assessment and early review of response to treatment, as detailed below.

### 2.3 1. Triage and information gathering

Initial triage should identify key risk factors (“red flags”) for sinister pathology and if present, the receiving team should consider whether rapid onward referral is appropriate. In the absence of endoscopic evaluation of the larynx (and lower airway), it is essential a low threshold is applied. Risks for onward referral includ ^16^:

- Persistent dysphonia
- Persistent noisy breathing
- Throat pain/ odynophagia
- Haemoptysis
- Smoking/ alcohol excess
- Unintentional weight loss

To assist MDT assessment, review of previous investigation data is vital. We recommend referrers provide as much existing data as available to support this (Table 1). If this does not occur, where possible, relevant information should be obtained preferably prior to triage, and at least prior to initial MDT review. Additional information that may be available and contribute to the assessment would be screening questionnaire data (eg Pittsburgh Vocal Cord Dysfunction Index; *21*), and any patient‐held (ie “selfie” type) video/ audio recordings made during symptomatic episodes. This may include recordings taken during exercise, providing this is conducted with patient safety appropriately considered.

**Table 1:** Suggested data set to facilitate triage/ MDT review. We acknowledge that different levels of data will be available dependent on the referral course (primary, secondary care) and may be particularly restricted if symptom onset occurred during the COVID‐19 pandemic

Presentation	Evidence of ILO (eg screening questionnaire data and patient‐held video/ audio recordings made during symptomatic episodes)
	Evaluation of co‐morbidities (eg asthma, reflux, nasal disease, voice disorder, cough and breathing pattern disorder)
	Drug history and current medications
Investigations	Physiology (eg spirometry ± reversibility, flow volume loop and exercise physiology)
	Data related to allergy (eg IgE, skin prick test results) and inflammation (eg blood/ sputum eosinophils and fractional exhaled nitric oxide)
	Imaging (HRCT scans, chest X‐rays)
	Endoscopy (bronchoscopy and laryngoscopy)
Correspondence	Copies of relevant speciality letters (in particular ENT, respiratory, gastroenterology and allergy)
	Historical questionnaire data [eg ACQ,^21^ VCDQ ^22^]

### 2.4 2. Assessment

If a patient has not had a respiratory review in the last 12 months this should occur prior to MDT assessment, with the aim of identifying other potentially treatable respiratory diseases that may be responsible for the presenting symptoms. MDT assessment and discussion of the likely diagnosis should follow, supported by the referral data obtained.

Specific consideration should be given to symptoms consistent with ILO but that may occur due to other factors. Emphasis on excluding uncontrolled asthma as a cause of breathing difficulties is a priority, but other common and important differential diagnoses to consider include breathing pattern disorders, and structural or inflammatory laryngeal and large airway pathology. This will prove challenging as access to lung physiology and bronchoscopy is also restricted.^13^, ^24^, ^26^ Surrogate measures such as peripheral eosinophilia and historic lung function should be used to guide and support this process.

Suitability for ILO intervention should be based on patients meeting the following criteria:

- Suspected ILO following MDT assessment and discussion;
- Suspected ILO likely cause of respiratory symptoms in patients with co‐existent disease (eg symptoms not attributed to breathing pattern disorder/ asthma);
- Other interventions not indicated (eg instrumental assessment/ physiotherapy for breathing pattern disorder);
- Patient consents to empirical SLT treatment, based on a presumptive ILO diagnosis.

At the time of publication, restrictions in performing respiratory instrumental assessments remain and even in a scenario whereby limited access to procedures becomes available, contingency plans need to be in place for further outbreaks or “second‐wave” surges. Consideration should be given to those patients deemed high priority for endoscopic evaluation (laryngoscopy ± bronchoscopy) as services begin to resume and access improves. It is likely that those patients identified as a high priority for laryngoscopy will similarly be a high priority for other objective tests (eg lung physiology). Even where resumption of laryngoscopy is possible, the throughput of patients will be significantly reduced due to mandated lengthy decontamination procedures between patients.

We have previously considered how to prioritize patients^27^ and recommend an adapted prioritization tool based on this document (Table 2). In the context of EILO, continuous laryngoscopy (CLE) may also identify abnormalities amenable to surgical intervention.^28^ Such management decisions are not possible without CLE and this approach will clearly be dependent on the broader background of surgical capability within the individual's service or health system territory.

**Table 2:** Guide to recommended prioritization (red, amber, green risk ratings) of patients for laryngoscopy (adapted from 24)

Priority	Selection criteria
High (acute) Risk rating = RED see Figure 1.	Urgent diagnosis required to prevent inappropriate tracheostomy, unnecessary ITU admission/intubation or to expedite discharge Severe, unrelenting known/ presumed ILO not responding to therapy
High (outpatient) Risk rating = RED see Figure 1.	Previous intubation due to suspected ILO Previous ITU admissions due to suspected ILO Frequent hospital admissions with suspected ILO High healthcare utilization Frequent courses of systemic corticosteroids, without expected response Significant patient distress Pre‐surgical upper airway assessment, including in the context of exercise‐ILO surgery (as appropriate)
Medium Risk rating = AMBER see Figure 1.	Symptoms have significant impact on daily function Frequent or severe ILO episodes Undergoing assessment for biological therapy for severe asthma, with high suspicion of ILO ILO with sequelae (eg high respiratory medication burden) ILO with associated significant dysphagia (where malignancy is not suspected)
Low Risk rating = GREEN see Figure 1.	Suspected ILO not meeting the above criteria

### 2.5 3. Early review of treatment response

Speech and language therapy is the gold‐standard treatment for ILO^29^ (in some centres, specialist physiotherapists deliver treatment based on SLT techniques). Laryngoscopy provides diagnostic confirmation of ILO, informs selection of the most appropriate SLT therapy techniques for symptom resolution and acts as a patient bio‐visual feedback tool. In the absence of endoscopic evaluation, SLT delivery is therefore an empirical treatment choice, but unguided by objective assessment.

Empirical SLT intervention (including symptom differentiation, laryngeal airway control techniques, reducing upper airway irritants and psychoeducational counselling) may not be appropriate for all patients. We recognize that approximately one‐quarter of patients with a high clinical suspicion of ILO do not have confirmed laryngoscopic appearances (*data on file*, based on over 300 referrals). Coupled with a reported non‐response rate of SLT intervention in endoscopically confirmed ILO patients of approximately 30%^25^ it suggests a non‐response rate to empirical treatment is likely to be high (approximately half of patients).

Considered review of treatment response is essential to minimize the risk of missing alternative pathologies and avoiding perpetuating an incorrect presumptive diagnosis of ILO. Objective sessional outcomes [eg clinician‐ and patient‐reported outcomes such as the Vocal Cord Dysfunction Questionnaire (VCDQ; *26*) and Newcastle Laryngeal Hypersensitivity Questionnaire^30^] should be recorded to monitor progress (using the “minimal clinically important difference” to grade response where available). Patients who fail to respond as expected, experience symptom deterioration or where the treating clinician has other concerns should be referred back to the MDT for discussion regarding ongoing management. It is important to note some patients may demonstrate fluctuating symptoms and therefore level of risk may change within the planned treatment period.

## 3 FUTURE STEPS

Recognizing the dynamic and unpredictable landscape that we are working in, the implementation of these recommendations should be audited, not only regarding their efficacy, but also their acceptability and feasibility for patients and healthcare professionals. Two key risks in particular should be recognized and monitored. The first relates to over‐treatment in the absence of the current gold standard; the second relates to under‐treatment for patients at risk of extreme distress, and high healthcare utilization.

Acceptability and feasibility should be monitored at a local level, recognizing variations in local resources, capacity and patient demographics. Service providers should recognize that patients often have different capabilities, or preferences for treatment place and type. Some may be hesitant about attending hospitals during the pandemic or lack confidence with tele‐medicine. We welcome the development of resources (eg videos) that can aid patient understanding of diagnosis and therapeutic interventions.

In the absence of routine laryngoscopy, the risk of misdiagnosis must not be overlooked and so it is imperative to maintain strong connections between respiratory and otolaryngology services. A research opportunity exists to investigate the diagnostic value of non‐invasive objective laryngeal assessments, such as dynamic CT, although this will clearly be currently limited by the difficulty of comparing to the gold‐standard laryngoscopy. Efforts should also be made to produce and refine validated outcome measures to better monitor therapeutic response.

Beyond the pandemic, lessons learned from workforce responses to clinical delivery, digital advances in tele‐medicine and further MDT integration patient management, present opportunities to establish and develop relationships between ILO experts across the globe. We look forward to greater collaboration to facilitate collection and amalgamation of larger data sets that will help us to further understand ILO and relative management strategies.

## Data Availability

Data sharing not applicable to this article as no datasets were generated or analysed during the current study.
